# Successful mitral valve reconstruction using autologous pericardium in a pregnant patient with severe infective endocarditis: a case report

**DOI:** 10.1186/s44215-025-00205-6

**Published:** 2025-04-03

**Authors:** Kimiaki Anai, Kazuki Mori, Takashi Shuto, Shinji Miyamoto

**Affiliations:** Department of Cardiovascular Surgery, Oita University, 1-1 Idaigaoka, Hasama, Yufu, Oita, 879-5593 Japan

**Keywords:** Infectious endocarditis, Mitral regurgitation, Pregnant patient, Autologous pericardium, Seamless reconstruction

## Abstract

**Background:**

A 34-year-old woman at 25 gestational weeks presented with severe respiratory distress secondary to heart failure caused by severe mitral regurgitation and infective endocarditis.

**Case presentation:**

Transthoracic echocardiography revealed a large (17 mm) vegetation attached to the posteromedial commissure of the mitral valve leaflet. Owing to pulmonary edema and circulatory failure, she underwent emergency cesarean section to improve maternal hemodynamics. Postoperatively, her pulmonary edema resolved, and the hemodynamic status was stable. Thus, mitral valve surgery was scheduled 2 days later. Intraoperative findings confirmed that the posteromedial site of the mitral valve was severely damaged by vegetation and chordae tendineae rupture. The damaged mitral valve leaflet was resected, with seamless reconstruction using a glutaraldehyde-fixed autologous pericardium. Postoperative echocardiogram revealed no residual mitral regurgitation. Despite premature birth, the infant survived but required surgery for patent ductus arteriosus.

**Conclusions:**

This case highlights that through timely intervention and advanced surgical techniques, a patient with severe infective endocarditis, despite being pregnant, can be successfully managed.

## Background

Infective endocarditis (IE) during pregnancy is a rare but life-threatening condition with significant maternal and fetal mortality. Despite advances in medical and surgical care, fetal complications resulting from IE remain a significant concern [1]. The optimal timing and approach for surgical intervention in pregnant women with IE are often challenging because both maternal and fetal well-being must be considered. Moreover, the choice of prosthetic valve affects future fertility, including the chances of pregnancy. This report presents a successful case of cesarean section (CS) and mitral valve repair for IE in a pregnant patient, highlighting the importance of timely intervention and careful surgical planning.

## Case presentation

**Fig. 1:**
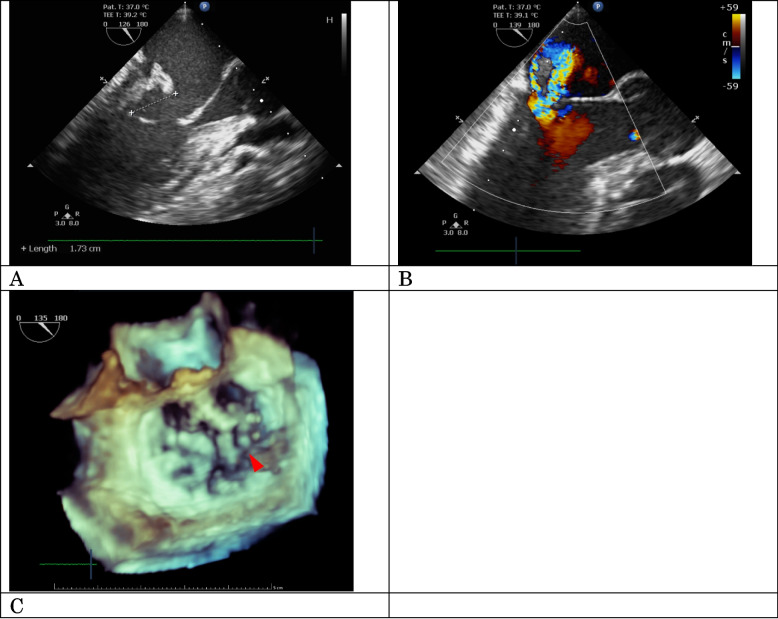
Preoperative echocardiographic findings. Echocardiography showed a 17-mm mobile mass at the posteromedial commissure mitral valve (**A**) and associated severe mitral regurgitation (**B**). Three-dimensional echocardiography revealed vegetation (arrow) on the mitral valve’s posteromedial site (**C**)

A 34-year-old woman, who was 7 months pregnant and had no significant medical history, including structural or functional cardiac disease, complained of abdominal pain. Antibiotic therapy was initiated with meropenem (1.5 g/day), but after 3 days, she developed orthopnea and acute pulmonary edema. She presented with an elevated white blood cell count of 12,190/µL and a C-reactive protein level of 7.34 mg/dL. Blood culture revealed *Streptococcus* *mitis/oralis* infection. In echocardiography, a 17-mm vegetation was noted on the posteromedial commissure of the mitral valve leaflet, consistent with the diagnosis of severe mitral regurgitation (MR) secondary to leaflet prolapse (Fig. 1).

Thus, the patient was urgently transferred to our hospital for surgical intervention. At admission, she was 25 weeks and 2 days pregnant. IE necessitated surgical intervention, but it could be detrimental to the fetus’ life; hence, a concurrent CS was considered. Given the patient’s desire for future pregnancies, a uterus-sparing approach was adopted. We then decided to perform an emergency CS first to improve the circulatory status, followed by cardiac surgery once the risk of postpartum hemorrhage had subsided.

Immediately upon her transfer to our hospital, we performed emergency CS. Venoarterial extracorporeal membrane oxygenation (VA-ECMO) was placed on standby for managing intraoperative hemodynamic instability caused by IE. Fortunately, the procedure was completed without VA-ECMO requirement. During the entire surgical course, her hemodynamic parameters remained within normal limits, and the total blood loss was minimal. After being intubated postoperatively, she was transferred to the intensive care unit (ICU), and her pulmonary congestion rapidly resolved.

**Fig. 2:**
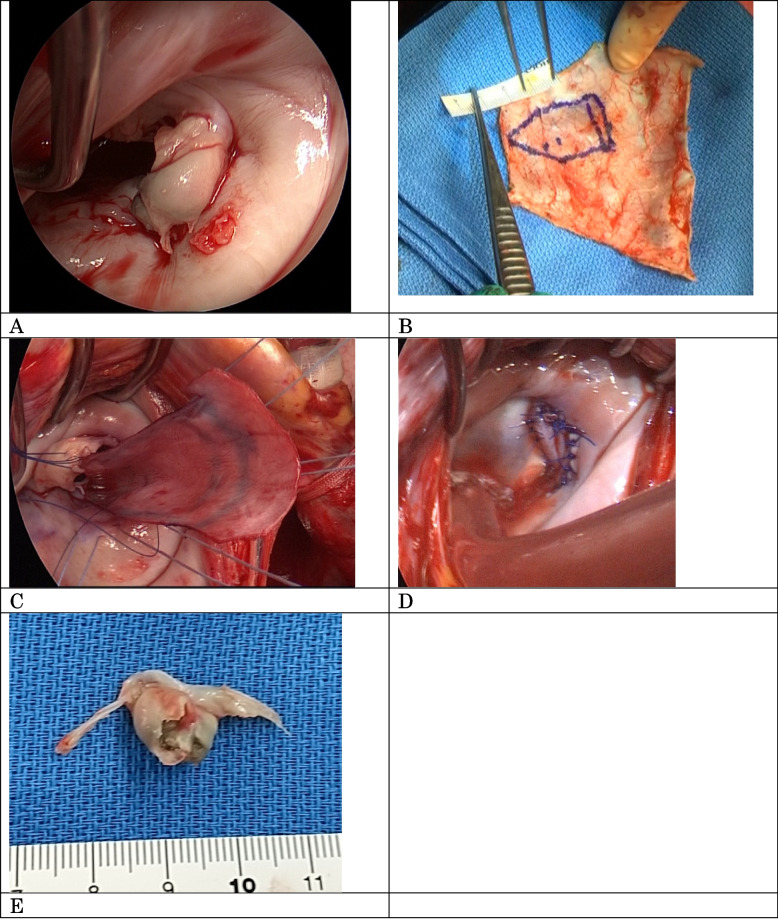
Intraoperative findings. A vegetation was attached to the mitral valve’s posteromedial leaflet on the ventricular side, extending from A3 to P3, with associated chordae tendineae rupture and leaflet prolapse (**A**). The annulus remained intact. Fixed autologous pericardium was shaped into a pentagonal patch (**B**). After the infected tissue was resected, a defect involving the A3–P3 region and commissure was created. The pericardial patch’s apical end was sutured to the posterior papillary muscle, and the lateral side was sutured to the resected mitral leaflet and mitral annulus (**C**). Water irrigation testing revealed no signs of mitral regurgitation and confirmed optimal valve geometry (**D**). Vegetation was observed on the left ventricular side of the excised mitral valve leaflet tissue (**E**)

**Fig. 3:**
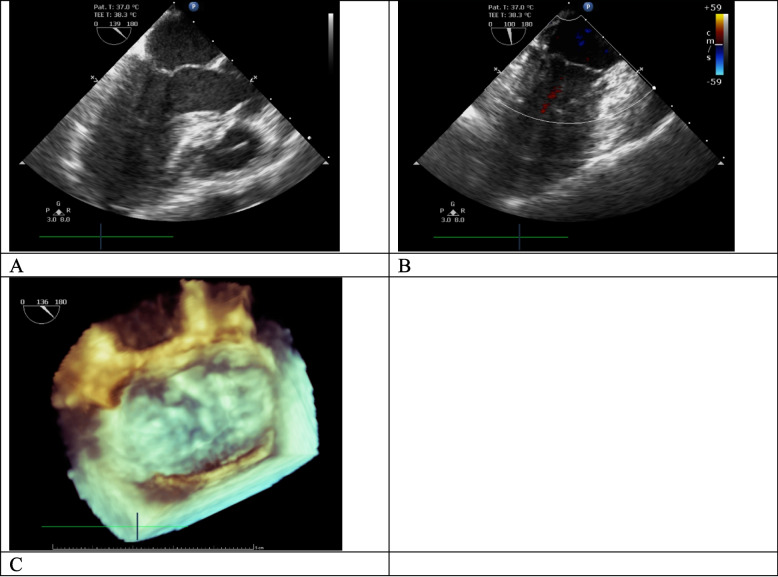
Postoperative echocardiography findings. Postoperative transesophageal echocardiography demonstrated successful mitral valve repair using the autologous pericardial patch (**A**), with no mitral regurgitation noted (**B**). The valve leaflets exhibited adequate coaptation during systole (**C**)

Two days after the CS, once the risk of postpartum hemorrhage had decreased, mitral valve repair was performed. A midline sternal incision was made, and the pericardium was widely harvested and fixed with 0.6% glutaraldehyde for 10 min. Subsequently, a cardiopulmonary bypass (CPB) was established. The mitral valve was reached through a left atrial lateral incision. A vegetation attached to the posteromedial mitral valve leaflets (A3–P3) on the left ventricle side was observed. Furthermore, this vegetation was found to rupture the chordae tendineae in that region. The infected vegetation and leaflets were both excised. The mitral annulus remained intact. The autologous pericardium, fixed in 0.6% glutaraldehyde, was shaped into a patch of pentagonal shape. The trimmed pericardial patch was sutured to the posterior papillary muscle using a 4–0 Prolene suture with pledget felt. To repair the leaflet defect, the surgeon sutured the leaflet and valve annulus to the patch using 5–0 and 4–0 Prolene interrupted sutures, respectively (Fig. 2). The operation lasted for 257 min, with 150 mL of blood lost. While the patient received heparin intraoperatively, no intraoperative genital bleeding occurred. She was extubated the day after surgery and was discharged from the ICU 2 days later. No intra-abdominal bleeding or intrauterine hematoma was observed in the following days. Moreover, ceftriaxone (4 g/day) was administered after cardiac surgery. Postoperative echocardiography revealed no residual MR (Fig. 3), and the patient’s postoperative course remained uneventful.

The infant, a male, was born extremely preterm, weighing 926 g with Apgar scores of 4 and 7 at 1 and 5 min, respectively. He was admitted to the neonatal intensive care unit (NICU), and at 12 days of age, he underwent ligation of patent ductus arteriosus (PDA). The infant gained weight steadily in the NICU and experienced no significant complications. Both the mother and infant were discharged in good condition.

After 1 year, the patient’s transthoracic echocardiography revealed trivial MR. She has remained free of anticoagulation and is in good health.

## Discussion and conclusions

The incidence of IE in pregnancy is approximately 1 in 100,000 pregnancies. Maternal mortality rates can reach 11–33%, whereas fetal mortality rates can be as high as 14–29% [1, 2]. Fetal survival rates significantly increase as the gestational age advances, particularly between 22 and 29 weeks. Therefore, balancing the competing goals of maternal survival and fetal viability is crucial when managing pregnant women with IE, especially at critical gestational ages, such as 25 weeks [3].

Surgical intervention is crucial for IE cases to prevent life-threatening complications, such as acute heart failure secondary to valvular dysfunction and embolic events, particularly stroke, caused by vegetation [4, 5]. Our patient presented with acute respiratory failure resulting from acute MR and eventually exhibited acute heart failure. Although embolic events had not occurred, the presence of vegetation exceeding 10 mm indicated a high risk of developing embolic complications, including stroke. Therefore, urgent surgical intervention was imperative.

Pregnancy complicates IE treatment; thus, a treatment plan that balances the need to preserve both maternal and fetal life is required. Reported surgical approaches for IE include CS followed by open-heart surgery within a few days, delivery after cardiac surgery, and concurrent CS and cardiac surgery [5–8]. Although our patient was at a high risk for embolic events because of vegetation, we opted for CS to save the fetus and alleviate acute heart failure. However, CS incision may put the patient at risk for excessive bleeding because of systemic heparinization during cardiac surgery. While total hysterectomy could mitigate this risk, the patient wanted to preserve fertility; hence, uterine-sparing surgery was performed. Fortunately, the patient successfully completed a 2-day waiting period for postpartum bleeding to subside, despite the ongoing risk of embolic events. During this time, her heart failure significantly improved. We have also encountered cases of IE during pregnancy treated with cardiac surgery, prioritizing maternal health because of fetal immaturity. Perioperative fetal monitoring and high temperature, high-flow CPB have been suggested to prevent fetal hypoperfusion during surgery [6, 7]. However, when maternal survival is at stake, pregnancy termination is the primary management strategy.

For pregnant patients with IE, individualized surgical strategies for valvular disease must be carefully considered. If the patient is young and has a recurrent infection risk, valve repair should be considered [4, 8]. However, the extent of infection often presents a significant challenge to repair. In our patient, resection of the infective lesion in the mitral valve resulted in a large A3–P3 defect, but the mitral annulus remained uninfected. Therefore, we successfully performed reconstruction using the autologous pericardium to seamlessly reconstruct the defective mitral valve and chordae [9]. The repair effectively controlled regurgitation with the use of a minimal prosthetic material without an annuloplasty ring. This approach is highly beneficial because it prevents recurrent infection and avoids the need for anticoagulation therapy. In cases of controlled heart failure, a strategy involving preoperative antibiotics for several weeks may be considered. This approach may optimize fetal growth and contribute to successful mitral valve repair by decreasing the amount of tissue excised.

In the case of mitral valve replacement, using biological or mechanical valves for IE has been controversial. A meta-analysis of observational studies found no difference in re-infection incidence, but the mechanical valve was superior the biological valve as it led to long-term survival and reduced reoperations [10]. Patients desiring to conceive in the future may consider the use of a bioprosthetic valve, and careful explanation of the long-term results to the patient would be important.

For pregnancies less than 25 weeks, the risks of premature birth and potential maternal and fetal complications of ongoing infection must be carefully balanced. Preterm infants born to these mothers often experience complications, such as low birth weight, low Apgar scores, respiratory distress syndrome, and intraventricular hemorrhage [3, 5]. Despite being born extremely preterm with low birth weight and Apgar scores, our patient’s infant successfully underwent PDA closure with a favorable outcome.

In our patient, preterm delivery was performed before cardiac surgery, resulting in successful fetal survival. Subsequently, our patient underwent successful mitral valve repair using the autologous pericardium, and the outcome was favorable. Management of IE during pregnancy requires a tailored treatment approach for each patient, considering their hemodynamic status, the presence of complications, and gestational age.

## Data Availability

Data sharing is not applicable to this article as no datasets were generated or analyzed during the current study.

## Abbreviations

CPB: Cardiopulmonary bypass
CS: Cesarean section
ICU: Intensive care unit
IE: Infective endocarditis
MR: Mitral regurgitation
NICU: Neonatal intensive care unit
PDA: Patent ductus arteriosus
VA-ECMO: Venoarterial extracorporeal membrane oxygenation
